# Effect of Resin Infiltration on Artificial Caries: An *in vitro* Evaluation of Resin Penetration and Microhardness

**DOI:** 10.5005/jp-journals-10005-1445

**Published:** 2017-02-27

**Authors:** Deepesh Prajapati, Rashmi Nayak, Deepika Pai, Nagraj Upadhya, Vipin K Bhaskar, Pujan Kamath

**Affiliations:** 1 Senior Lecturer, Department of Pedodontics, NIMS Dental College and Hospital Jaipur, Rajasthan, India; 2 Professor and Head, Department of Pedodontics and Preventive Dentistry, Manipal College of Dental Sciences, Manipal, Karnataka, India; 3 Reader, Department of Pedodontics and Preventive Dentistry, Manipal College of Dental Sciences, Manipal, Karnataka, India; 4 Associate Professor, Department of Dental Materials, Manipal College of Dental Sciences, Manipal, Karnataka, India; 5 Senior Lecturer, Department of Pedodontics and Preventive Dentistry, Mahe Institute of Dental Sciences & Hospital, Marie, Puducherry, India; 6 Private Practitioner, Department of Pedodontics and Preventive Dentistry, Manipal College of Dental Sciences, Manipal, Karnataka, India

**Keywords:** Dental caries, Dental materials, Microhardness, Resin infiltration.

## Abstract

**Aim:**

To evaluate the effectiveness of resin infiltration on artificial caries lesion by assessing the depth of resin penetration and the change in microhardness of lesion postinfiltration.

**Materials and methods:**

Totally 45 human extracted premolars were used to create an artificial demineralized lesion in enamel using demineralizing solution. A total of 15 samples (group I) were infiltrated with resin. The depth of resin penetration was studied using scanning electron microscope (SEM). Other half (n = 30) of samples was equally divided into three subgroups and Vickers hardness number (VHN) values were obtained to measure the surface microhardness as group 11 a—before demineralization, 11 b—after demineralization, IIc—postresin infiltration.

**Results:**

Mean depth of penetration in group I was 516.8 urn. There was statistically significant increase in VHN values of demineralized lesion postresin infiltration (independent Student’s t-test, p < 0.001).

**Conclusion:**

Penetration depth of the resin infiltrant was deep enough to render beneficial effects, while significant increase in microhardness was observed postresin infiltration.

**Clinical significance:**

Infiltrant used can be considered as a valid treatment option for noncavitated lesions.

**How to cite this article:**

Prajapati D, Nayak R, Pai D, Upadhya N, Bhaskar VK, Kamath P. Effect of Resin Infiltration on Artificial Caries: An *in vitro* Evaluation of Resin Penetration and Microhardness. Int J Clin Pediatr Dent 2017;10(3):250-256.

## INTRODUCTION

Different approaches to caries removal have been attempted through the years, starting from the use of a hand drill, which was surpassed in 1871 by James Morison’s treadle instrument.^1^ Even today, conventional caries treatment usually involves use of a highspeed handpiece to access the lesion and a low-speed handpiece to remove the caries, i.e., predominantly an invasive approach. This approach removes the carious tooth structure along with unintentional removal of noncarious tooth structure.

Minimally invasive cavity designs and techniques have also been tried in an effort to minimize the amount of destruction due to tooth preparation like air abrasion, atraumatic restorative therapy, chemomechanical caries removal, and lasers. Nonetheless, minimally invasive approach is late in the disease process and destructive as well, resulting in loss of the original anatomy, strength, and esthetics and, thereby, leading to continuum of replacement dentistry^2^ or referred to as the "death spiral of restorations".^3^

In recent decades, a much more tissue-preserving approach to arrest and control proximal or smooth surface carious lesions has been studied extensively, namely resin infiltration. This concept aims at occluding the highly porous structures of incipient enamel lesion by means of low-viscosity resins. This approach truly follows the principles of minimally invasive dentistry, one which is scientifically oriented, helps in diagnosis of early carious lesions using diagnostic devices and minimal surgical intervention of cavitated lesions.

Resin infiltration offers advantages like mechanical stabilization of the demineralized lesion, preservation of sound hard substance (protection of both the same and the adjacent tooth), permanent occlusion of the superficial micropores and cavities, arrest of lesion progression, minimized risk of secondary caries, delay of restorative intervention for longer periods, no risk of postoperative sensitivity and pulpal inflammation, reduced risk of gingivitis and periodontitis, improved esthetic outcome when used as a masking resin on the demineralized labial surfaces (white spot lesion, in orthodontic patient), and high patient acceptance.

Many materials have been tried for infiltration in the past. One such material—Icon—the caries infiltration product was introduced in Germany in 2009. This product utilizes a special resin to fill and seal diseased enamel, with no unnecessary loss of healthy hard tissue. Manufacturers claim that this is an innovative product for the microinvasive treatment of early carious lesions in the approximal and vestibular regions. It can be used to treat caries in a timely manner without drilling. The approximal version of the product is specially developed for hard tissues, preserving treatment of incipient proximal caries; the vestibular version is particularly suited for orthodontic patients after braces removal.

To our knowledge, only few studies have been conducted regarding resin infiltration using any commercially available infiltrant and they have shown to have promising results. Knowing about the advantages this technique offers and lack of studies in this area, it warrants extensive research to formulate evidence-based guidelines for the same.

## MATERIALS AND METHODS

This *in vitro* study was conducted with the aim to evaluate effectiveness of resin infiltration on artificial caries lesion. The objectives of the study were to assess the depth of penetration of resin into artificial caries lesion and assess the microhardness of lesion postinfiltration and compare it with that of normal and demineralized enamel. Null hypothesis was resin infiltration does not alter the micro-hardness of artificial carious lesion.

The study was initiated after obtaining ethical committee approval from the ethical committee (IEC 10/2011). The ICON caries infiltrant (DMG, Germany) with batch no. 634903 was used in the present study.

A total of 45 noncarious premolars with sound coronal structure, which were extracted for orthodontic purpose, were obtained. Teeth with hypoplasia or incipient carious lesions/white spots were excluded. These teeth were stored below room temperature in normal saline until they were subjected to any intervention. The selected teeth were divided among two groups as shown in Flow Chart 1.

### Production of Artificial Demineralized Lesion

Totally, 45 teeth were coated with nail varnish (Street Wear, nail color, Modi-Revelon Pvt Ltd) after sticking an adhesive tape of 5 mm by 3 mm size on labial surfaces of each tooth. Once the nail varnish was dry, the adhesive tape was removed, which resulted in a window of the same size, where demineralization was to be produced.

**Flow Chart 1:**
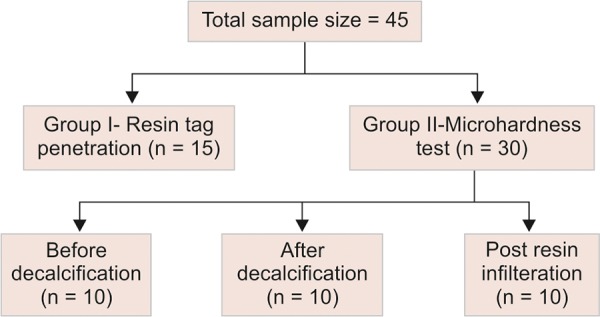
Division of samples in various groups

Demineralizing solution was prepared using nominally 100 mmol/L lactic acid, 18 mmol/L calcium chloride, 7.8 mmol/L monobasic potassium phosphate, and 3 mmol/L sodium azide as a bacteriostat.^4^

About 35 of the prepared teeth were now dipped in demineralizing solution to produce incipient enamel caries lesion. A pilot study was done to determine the depth of demineralization after placing the sample in solution for 21 days. Each tooth was submerged in sterile plastic containers containing 50 mL of demineralizing solution for 21 days. The pH of demineralizing solution was checked every day and was maintained at 4.3 with potassium hydroxide.

About 5 of 15 samples from group I were randomly selected for SEM analysis and a sample size of 30 was decided for the microhardness group. Since individualized variations are minimum in *in vitro* samples as compared with human subjects, the above-mentioned sample sizes for both groups were selected.

### Resin Infiltration

Each carious lesion produced was treated with resin infiltration. The application of resin was done as follows: 15% HCl was applied for 2 minutes on the lesion, and then etchant was rinsed for 30 seconds and air dried. About 99% ethanol was applied for 30 seconds and air dried followed by resin infiltrant (methylmethacrylate) application, which was left on the lesion for 3 minutes before curing. This was light cured for 40 seconds. The application of resin was repeated for 1 minute and again light cured for 40 seconds.

### Scanning Electron Microscopy

Randomly selected 5 samples of group I were embedded in self-cure acrylic blocks to facilitate sectioning it in buccolingual plane, using hard tissue microtome (Leica 1600). These blocks were then subjected to drying process by placing them in hot air oven (Heathron Industrial Heaters) for 10 minutes at 110°C. Samples were then ready to be subjected to SEM (JEOL, JSM-6380LA) to investigate the depth of penetration of the infiltrant in this group.

**Fig. 1:**
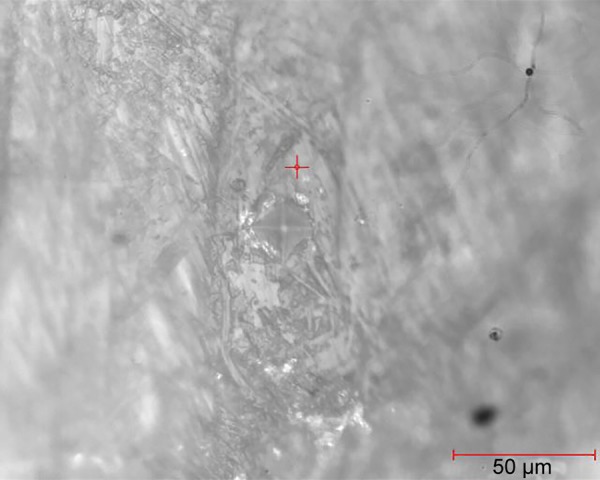
Indentation on one of the samples of normal enamel

**Fig. 2:**
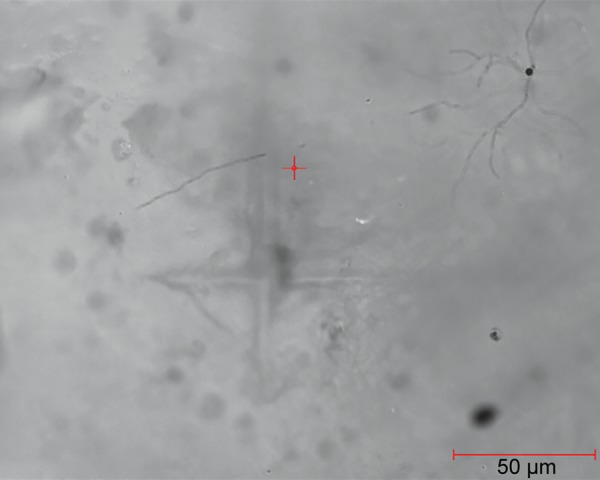
Indentation on one of the samples of demineralized enamel

The samples were mounted in the platform after covering them with nonconducting tape. These taped samples were subjected to gold sputtering (JEOL JFC-1600 Auto Fine Coater) in order to facilitate conduction of electricity, as enamel is considered a bad conductor of electricity and then examined under SEM for penetration depth of the infiltrant resin into artificial caries lesions.

**Fig. 3:**
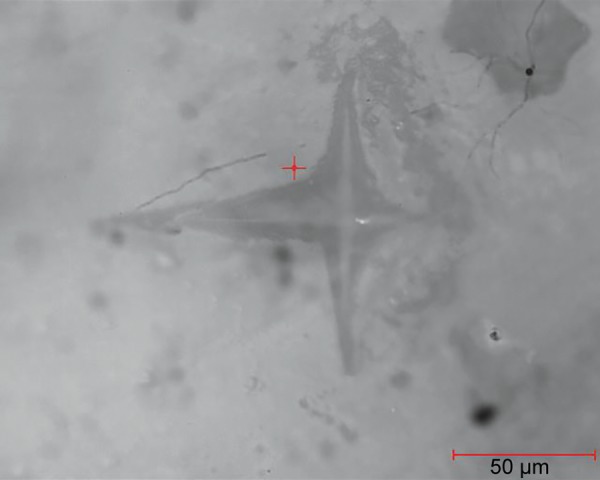
Indentation on one of the samples postinfiltration

### Surface Microhardness Evaluation

Vickers microhardness tester (CLEMEX CMT HD) was used to investigate the change in hardness postinfiltration. At baseline 10 non decalcified teeth of group IIa were tested for microhardness of enamel. The values were also measured after decalcification and compared with the values postinfiltration.

For the microhardness testing, a load of 100 gm was applied for 10 seconds as recommended by Chuenarrom et al.^5^ Five indentations, at least 100 μm apart, were made at the center of each specimen and then were averaged.

Since the hardness test requires the testing surface to be flat and parallel to the base, the teeth were mounted first on acrylic after creating the window. Care was taken to see that the crown was projected thus, ensuring that the convex smooth surfaces were as parallel to scanning stage as possible. These teeth were then cut using carborundum disk to make the top layer flat and later polished using a circular grinding machine from 500, 800, 1000, 1200 up to 4000 grit (silicone carbide abrasive papers). Samples were prepared in accordance to the study done by Toledano et al.^6^

The samples were placed on the scanning stage and observed under microscope of 400x magnification for flattest surface available to indent. To ensure accuracy of the measurements, indentations were done on the flattest points of the enamel surface. This flattest point on the testing surface was determined using the microscope attached along with the microhardness tester at 400x magnification.

After selecting the surface, the indent was made and was again observed under microscope for measuring the diagonals of the indentation under same magnification. These diagonals were marked using the VHN software and, hence, the VHN values were determined. Figures 1 to 3 show the microhardness tester indentations in different groups.

Data were entered in data spread sheet using Statistical Package for the Social Sciences version 14 software. Independent sample t-test was used for group II to obtain the results.

## RESULTS

**Fig. 4:**
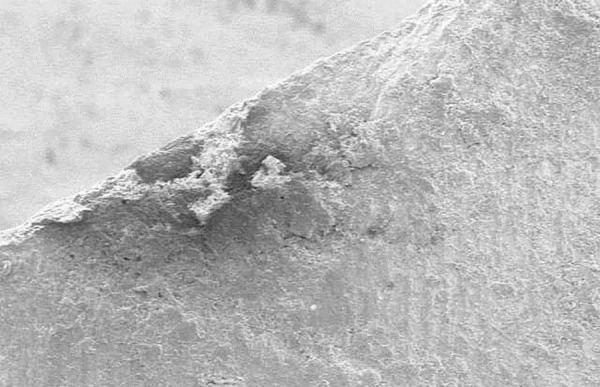
Pilot study sample with decalcification (average depth was 341 μm)

At preliminary stages, the mean depth of demineraliza-tion of 341 μm (Fig. 4) was established in pilot samples, by subjecting them to the demineralization solution (for 21 days) as decided under methodology.

**Fig. 5:**
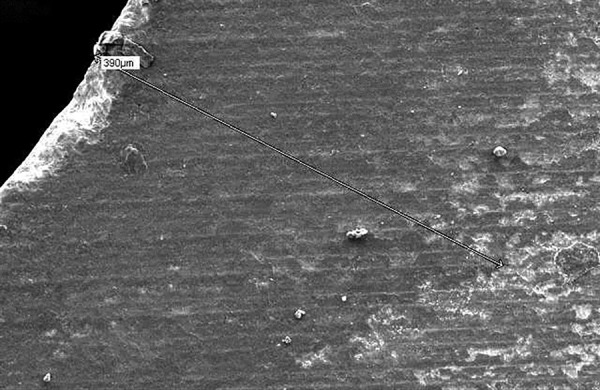
Scanning electron microscope view with 390 μm depth of resin tag penetration on demineralized enamel of a sample at 250* magnification

**Fig. 6:**
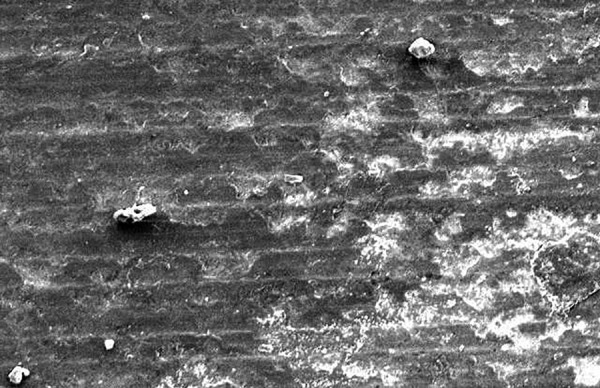
Scanning electron microscope view with junction of demineralized enamel and infiltrated resin in a sample at 500* magnification

Inverted cone appearance in the test samples was interpreted as demineralized zone. The SEM observation of resin infiltration samples showed resin infiltrated tags. The depth of infiltration thus analyzed up to deepest point ranged from 353 to 973 μm in group I (Table 1). Figures 5 and 6 show the depth of infiltration in one of the samples from group I at two different magnifications of 250x and 500x.

Vickers microhardness tester was used to determine the change in hardness of the artificial demineralized lesions post resin infiltration. Table 2 shows the VHN values for group II and Table 3 represents the mean values of microhardness for each subgroup. Intergroup comparison was done between groups IIb and IIc using independent sample t-test. This gave a statistically significant increase in hardness post resin infiltration (p < 0.001). Thus, deriving that resin infiltration does significantly affect the surface microhardness of the demineralized lesions. Figures 1 to 3 show the indentations of one sample each for all three subgroups at 400x magnification.

**Table 1:** **Table 1:** Depth of penetration of resin tags

*Sample no.*		*Penetration depth without thermocycling (μm)*	
1		353	
2		390	
3		401	
4		973	
5		467	

**Table 2:** **Table 2:** Mean of five VHN values of each of the samples

*Sample no.*		*Predecalcification (group lla)*		*Postdecalcification (group llb)*		*Postinfiltration (group llc)*	
1		262.2		5.2		13	
2		314.6		4.4		15.4	
3		285.6		6.2		24	
4		290.8		5.4		10.2	
5		285.2		6.2		9	
6		386.8		9		9.4	
7		319.2		3.8		9.4	
8		358.2		5.4		10.2	
9		234.2		7.8		10.4	
10		242.2		5.6		10.2	

**Table 3:** **Table 3:** Mean of the VHN values

*Groups*		*Mean± SD*			
IIa		234.2 *±* 48.38		p = 0.001	
IIa		5.9 *±* 1.53		(comparing IIb and	
IIc		12.12 *±* 4.6		IIc groups) HS	

HS: Highly significant; SD: Standard deviation

**Fig. 7:**
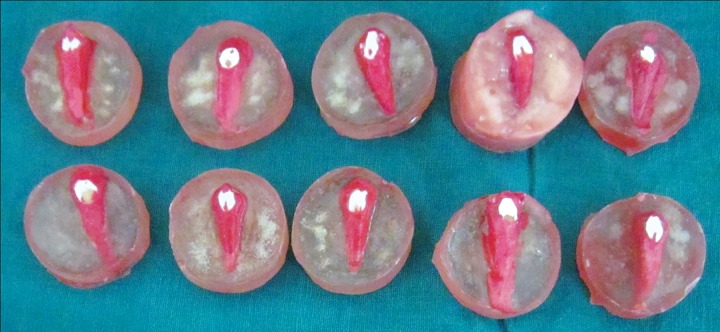
Samples after decalcification

**Fig. 8:**
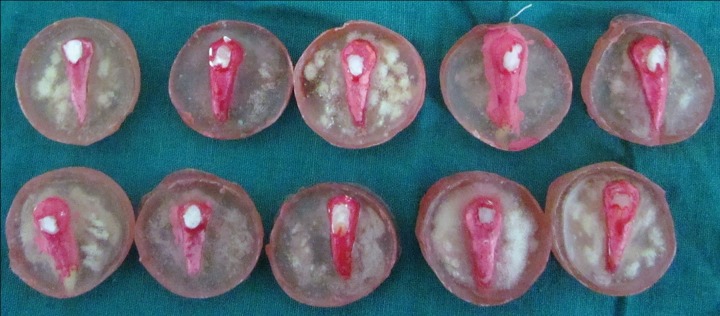
Samples postresin infiltration with reversal of enamel translucency to normal

There was an obvious change in the color of the enamel in the window after decalcification, which reverts back to predecalcification translucency of enamel after resin infiltration (Figs 7 and 8). Quantifying or studying this color change was not the objective of this study and, hence, was not attempted.

## DISCUSSION

Arresting enamel lesions by infiltration with resins is a promising approach for the nonoperative treatment of incipient enamel carious lesions.^7^ The concept of resin infiltration aims at arresting the incipient enamel caries lesions by obstructing the diffusion pathways for acids and dissolved minerals in enamel.^8^ An infiltrant resin used for this purpose must possess very low viscosity, a high surface tension, and a low contact angle with the enamel, all of which are important properties for penetration of the resin into the body of an incipient enamel lesion.^9^

Many demineralizing agents have been used to produce *in vitro* enamel demineralization models for example, acetic acid,^10^ lactic acid,^4^ or acidified hydroxy-ethylcellulose system.^11^ A formulation containing lactic acid was selected for our study due to easy availability of all the ingredients. A preliminary SEM observation of a demineralized sample was made to ascertain if satisfactory demineralization could be achieved with the planned formulation prior to the start of the study. This step was essential because satisfactory demineralization was an important prerequisite for studying resin infiltration *in vitro.*

In the present study, 15% HCl was used for conditioning of the lesion surface. The hypermineralized surface layer of an incipient enamel lesion impedes penetration of the resin into the lesion body. Thus, the aim of the conditioning procedure is to completely remove the surface layer and expose the lesion body. Resin penetration depths ranging from 10 to 500 μm have been reported by previous studies which used 15% HCl as the surface conditioner.^12^ Paris et al^9^ compared the penetration depth following conditioning with 37% phosphoric acid or 15% HCl and concluded that etching with 15% HCl gel is more suitable than 37% phosphoric acid gel as a pretreatment for caries lesions intended to be infiltrated. Meyer-Lueckel et al^12^ reported that 15% HCl removes approximately 40 μm of the hypermineralized surface layer in contrast to enamel microabrasion, which removes up to 360 μm of enamel.

It is essential to remove the water, i.e., stored inside the microporosities of the lesion body and then allow the resin to penetrate into it, which is driven by capillary forces.^13^ About 99% ethanol used in our study achieved this effect.

Following surface conditioning and dehydration, the infiltrant resin was applied twice over the lesion in order to occlude the space generated by the shrinkage of material after the first application.^14^ The application time was kept at 3 minutes according to manufacturer’s instructions.

It is a well-known and accepted fact that clinical conditions cannot be reproduced in *in vitro* conditions. Proper infiltration depends on excellent moisture control, which is undoubtedly easier in case of *in vitro* studies. It is possible that higher penetration depths are obtained *in vitro* compared with *in vivo* conditions. Hence, the results of this study must be carefully interpreted.

In this study, the SEM was used to determine the depth of penetration of the infiltrant resin. A pioneering work done by Hubbard^15^ on surface changes of artificial carious lesion with SEM and its correlation with light microscopic observations with the help of a photographic tracing procedure concluded that SEM provided useful information about the structural change occurring at the rod and crystalline levels in artificial carious lesion. His findings also correlated to the distinctive zonal patterns observed by light microscope and also provided support to the concept of dynamic nature of caries process. Saviero et al^16^ also used SEM images in their study, which was conducted to assess the resin infiltration depth in lesion produced *ex vivo.* Hence, SEM was used in this study, which is in accordance with other authors who conducted similar studies.^16^

Early enamel lesions (white spot) have already been proven to have lower microhardness than intact caries-free enamel surface.^17^ Knowledge of the mechanical properties of human enamel and dentin under normal and altered conditions would help restorative treatment.^18^ In general, restorations prefer to be retained by hard tooth structure for better mechanical stability.^19^ It is postulated that the infiltrated resin matrix can also strengthen the enamel structure mechanically.^14^ In an unpublished study by Palamara et al,^20^ it was observed that microhardness also can be a parameter to detect the change in mineral content of the lesion. Therefore, an attempt was made to study the change in microhardness of infiltrated lesions in our study.

Microhardness tests are widely used to measure the hardness of teeth.^5^ This method is easy, quick, and requires only a tiny area of specimen surface for testing. Gutierrez-Salazar and Reyes-Gasga in 2003 recommended that Vickers hardness tests must be preferred for tooth hardness testing as against the Knoop hardness tests. Accordingly, Vickers hardness test was preferred for this study.

Previous studies have used different indenters and various loads and times to investigate the hardness of enamel and dentin.^6^ In the present study, the specimen surfaces were impressed with a diamond indenter at a load of 100 gm for 10 seconds. The lowest load of 100 gm for enamel was selected for this study because it created Vicker’s diagonals longer than 20 μm, which is recommended to prevent errors in optical measurement.^21^ A study done by Chuenarrom et al^5^ studied the effect of indentation load and time on Knoop and Vicker’s micro-hardness tests for enamel and dentin; it showed that the difference of loading times (10, 20, and 30 seconds) was not significant for either enamel or dentin tested for the same test load. This suggested that an indentation time of 10 seconds is sufficient for a permanent indentation on the tooth surface to take place.^5^ Accordingly, an indentation time of 10 seconds was used in the present study.

The microhardness of resin-infiltrated enamel lesions demonstrated an improvement, but it did not rise to the predecalcification levels, suggesting that the resin infiltration technique may not be able to bring back the original microhardness levels. However, according to Torres et al,^22^ Kim et al,^23^ Palamara et al,^20^ it was observed that the microhardness of carious lesions increased with the infiltration of resin.

Postresin infiltration VHN values were found to be significantly greater than postdecalcification values. Predecalcification values being clearly greater than the values in other two groups did not warrant any statistical test.

An artificial *in vitro* enamel lesion model was used in this study, which limits the external validity of the study because under clinical situations, the lesions to be resin infiltrated are deeper.^24^ Hence, more studies are needed to confirm the efficacy of resin infiltration techniques under clinical conditions.

## CONCLUSION

Following conclusions can be drawn from the study:

- Resin infiltration technique is an upcoming microinvasive approach for management of noncavitated lesions and is expected to increase the span of microinvasive dentistry.
- Surface microhardness of infiltrated enamel lesions increases significantly. However predecalcification hardness values cannot be achieved, suggesting that the original hardness of enamel will not be regained by resin infiltration.

## CLINICAL SIGNIFICANCE

Infiltrant used can be considered as valid treatment option for noncavitated lesions.
